# The Structure of the Concepts Related to Love Spectrum: Emotional Verbal Fluency Technique Application, Initial Psychometrics, and Its Validation

**DOI:** 10.1007/s10936-019-09661-y

**Published:** 2019-08-09

**Authors:** Barbara Gawda

**Affiliations:** Department of Psychology of Emotion and Cognition, Institute of Psychology, Maria Curie-Skłodowska University, Plac Litewski 5, 20-080 Lublin, Poland

**Keywords:** Emotional verbal fluency, Love, Emotion concepts, Cognitive structure, Method, Psychometric properties

## Abstract

Love has been thoroughly studied and a variety of definitions as well as types of love have been described in the literature. Given the data presenting natural language of love concept, the aim of the two present studies is to demonstrate a new technique which enables description of the structure of emotion concepts within love spectrum. This technique is based on emotional verbal fluency tasks. The procedure and the coding system used are reported in the article along with the data on reliability and standardization of the emotional verbal fluency technique. Construct validity is demonstrated by correlations of the emotional verbal fluency tasks with semantic and letter verbal fluency tasks, while discriminant validity is shown by correlations with other measures, such as the Triangular Love Scale, the Love Attitudes Scale, and the State-Trait Anxiety Inventory. The article also presents how the emotional verbal fluency technique can be used in exploring the structure of emotion concepts within love spectrum. This is based on a hierarchical cluster analysis. An examination of connections between semantic clusters identified in the emotional verbal fluency tasks allows describing a structure of the concepts within love spectrum, such as *liking, infatuation, love*, and show of its differentiation. The presented technique taking into account natural language is of value in assessments of the structure of emotion concepts.

## Introduction

### Emotional Concepts

Generally, concepts are forms of mental representation of a cognitive set of objects, whether natural, artificial or hypothetical (Eysenck 2012). We can say that these are sets of features related in meaning or function. There are two basic groups of concepts derived from the types of categorization: classical concepts and natural concepts (Eysenck 2012). The former are related to the theory of classical concepts and are based on an empiricist approach or a standard set theory assuming that it is possible to unambiguously determine the belonging of a given concept to a category where there are strict limits and binary attributes (Fehr and Russell 1991). Classical concepts are thought to be clearly defined, with clear definitional boundaries, and definitional structure. In turn, natural concepts are less precise, they have a more or less specific character. This type of concepts is derived from a trend called realism and is closely related to the theory of the prototype (e.g. Medin 1989). Natural concepts are dependent on changes brought about by experience and an impact of the situation. The construction of natural concepts explains the theory of fuzzy sets, and these concepts are elements of lay-representations of the world (Fehr 1988, 2005). This group includes emotional concepts. They will be understood as concepts that, to varying degrees, denote emotions such as *love, liking*; these are not words with emotional connotations such as *dog, tree*. The studies were focused on the concepts within the basic emotion spectrum, i.e. *lov*e is a basic emotion while *liking, infatuation, passion* are terms within love spectrum. These terms denote an emotion of love, they are emotional words but they do not carry an emotional connotation. Emotional concepts according to Niedenthal (2008, p. 587) are mental representations of categories, objects, situations, and activities. Emotional concepts contain information about the causes of emotions, i.e. situational factors potentially causing emotions, typical behaviors or activities corresponding to a given emotion, and internal subjective states typical for a given emotion, and its meaning (Niedenthal 2008, p. 588). In general, conceptualization of emotions is characterized by several properties: unfocused boundaries, existence of bipolar dimensions in some cases, category relativity, typology, existence of intercategorical relationships, linking with scripts and scenarios, hierarchical organization and rooting in more general theory of mind (Russell and Bullock 1986). A description of natural concepts, such as emotional concepts, requires a reference to natural/lay language (Wierzbicka 1992a, b). Language analysis enables description of mental representations in a form of concepts, because language is a part of the mind and simultaneously a phenomenon that constructs human mental and social capabilities (Wierzbicka 1992a). The best known models of emotional concepts formulated by scientists include dimensional, semantic units, prototype, and semantic network models (Niedenthal 2008; Niedenthal et al. 2006).

Another conception was formulated by Russell (1991, 2005) who proposed a concept of emotion as a script. Emotional concepts are events that have a causal and temporal structure. The concept of the script describes the structure of emotion concept, also taking into account prototype elements. Its structure includes key elements related to emotional situations more specifically such factors as: causes, beliefs, sensations, physiological changes, desires, activity, as well as vocal, and mimic expressions. The assumption about the prototype of affective concepts has been confirmed in many studies using material with different modalities, requiring recognition and naming of emotions or narratives. However, it has been shown that some emotion concepts are characterized by a distinct structural identity, e.g. they contain a list of features or unique specimens (according to the exemplar theory: Kroska and Goldstone 1996).

The aim of the current study is to present a new way of assessing the structure of emotion concept. And the example of this is a love concept.

### Love: A Differentiated Emotion and a Differentiated Concept

Love is defined in the literature in a variety of ways: as a relationship, as an attitude, as an experience, and as an emotion (Fehr and Russell 1991). This complex emotion consists of affective experiences with positive valence, for instance joy, elation, sexual arousal, contentment, delight, a sense of happiness (Fehr 2006). Although positive valence dominates in this group of feelings, negative elements which may appear include anxiety, fear, dissatisfaction, jealousy, hatred, shame or guilt (Sternberg 1986). In the literature it has been emphasized that this emotion is not uniform in terms of the structure of affective feelings, the type of object to which the feeling is addressed, and the phase of relationship (Sternberg and Weiss 2006; Hendrick and Hendrick 2006). Furthermore, there are many other determinants influencing this complex emotion (Dion and Dion 1996, 2006; Hatfield et al. 2007; Schmitt et al. 2009). Researchers exploring romantic love have established that the differentiation of understanding and experiencing of love is determined by a huge number of factors (Shiota et al. 2010; Smith and Klases 2016; Sternberg and Weis 2006). A crucial role in this respect is attributed to the factors of gender, age, personality traits, individual experiences, and emotional maturity (Schmitt 2006). There are several theories of love proposed in psychological literature that have been comprehensively described, e.g. in the book entitled *New psychology of love*. One of the best known is the three-factor conception of love, formulated by R. Sternberg, assuming there are three components of love: intimacy, passion, commitment. These components evolve over time and are constantly changing (Sternberg 1986). Another conception by Sternberg defines love as an individual story that a person continuously builds in the course of development and experiences. It is a narrative concept containing basic generalized information about love. The nature of this narrative has a great impact on the quality and type of a specific person’s relationships. People construct different stories of love, and their abstractive versions are stored in memory forming a representation of affective experiences. The structure of this representation is hierarchical (Sternberg 1998, 2006).

Fromm’s (1956) conception of love, for instance, assumes that love is a character property, an individual’s attitude towards the world, and not to one person only. The theory by Hatfield (Lieberman and Hatfield 2006) highlights the existence of two basic types of love, passionate and companionate. Passionate love can be defined as a state of intense desire to unite with another person (Landis and O’Shea 2000). Companionate love is defined as sympathy and tenderness towards people with whom our lives are related. This kind of love is characterized by a very close friendship, sympathy, respect, mutual concern, and attraction (Fehr 2006; Hatfield and Rapson 1987, 1996; Kim and Hatfield 2004). Another conception of love formulated by Lee (Hendrick and Hendrick 1986, 2006) distinguishes six types of love, by reference to the Hellenic ideals of Eros, Storge, Ludus, Pragma, Mania, and Agape. The lay-conception of love described by Fehr points out that most people associate love with honesty, trust and caring, and rarely with addiction, sexual passion, or physical attractiveness. Fehr (2006) argues that lay-people have quite extensive knowledge about the concept of love. The lay-concept of love includes both the properties of companionate and passionate love. The most prototype features of love are trust, caring, and intimacy, whereas secondary features of love include staring at the other person, sexual passion or fast heartbeat. This means that the features of companionate love are considered to be more prototypical features of lay-concept love. Personal lay-conceptualization of love is associated with personality traits. For instance, people displaying high measure of protectiveness have a propensity for companionate love, while more dominant people for passionate love (Fehr 2005, 2006; Fehr and Broughton 2001; Fehr and Russell 1991).

As regards the concept of love, researchers have established that this concept has a prototype construction (Aron and Westbay 1996; Fehr 1988; Fehr and Russell 1991). Shaver and associates described the prototype structure of love taking into account words with emotional connotations. They have shown that love is basic/fundamental among emotional concepts. The structure of love concept includes factors preceding the occurrence of love, such as an atmosphere of openness, a sense of security, then an emotional mood: the relationship between people based on a profound feeling that they are liked, loved, need each other; it also incorporates activities: there is a very good compatibility between the people. As regards the manifestations of love, cognitive aspects are discussed as well, for instance difficulties with concentration, and attention focusing on a beloved person. Then, emotional aspects have been highlighted, e.g. a strong feeling of happiness and joy, and a sequence of behaviors expressing a need to be together. The components of the concept of love suggest that the concept of companionate love prevails in the whole structure of love (Shaver et al. 1987; Sternberg and Weis 2006). Elements of passionate love are definitely less intense in this structure, although they are also marked in the search for physical closeness, desire, and so on (Hatfield and Rapson 1987, 1996; Shaver et al. 1987).

The present research program was designed to explore the structure of the concepts representing the spectrum of love, taking into account linguistic material. This approach was based on the assumptions that language analysis can be of value in describing the semantic structure of emotional concepts (drawing on the thesis that “language is a tool used to express meanings”, as proposed by Wierzbicka 1996, p. 19; when we explore a language we refer to meanings which are a way of understanding something by a person).

### Emotional Verbal Fluency

This paper addresses the problem of cognitive structure/internal organization of the emotional concepts from the viewpoint of semantic relationships expressed through emotional verbal fluency. Verbal fluency techniques belong to the classical psychological tools used in the diagnosis of cognitive functions, mainly in neuropsychology (Ardila and Ostrosky-Solí 2006). Verbal fluency techniques generally rely on spontaneous listing of words according to a criterion. Therefore, the subject is asked to name words representing a given category/criterion within a certain amount of time (usually 1 min) (Gawda and Szepietowska 2013, 2016). Apart from the standard applications of verbal fluency techniques, it was assumed that by using qualitative-quantitative analysis of words generated during emotional verbal fluency tasks, it would be possible to describe the organization and structure of emotional concepts. This approach is justified by the evidence showing that this technique enables effective exploration of verbal, episodic, and semantic memory (Goni et al. 2010). To our knowledge, few results based on the use of such a methodology have been published, and traditional indicators, such as the number of correct responses, errors, semantic switches, semantic clusters, phonemic switches, and phonemic clusters have been taken into consideration in the analyses; typical analysis usually consists in quantifying the total production of words (e.g. Abeare et al. 2017; Gawda and Szepietowska 2013, 2016; Rossell 2006; Sass et al. 2013; Wauters and Marquardt 2017).

Emotional/affective verbal fluency (EVF) is a novel and rarely used semantic fluency type which can take a variety of forms: positive versus negative, pleasant versus non-pleasant, joy versus fear, etc. (Gawda and Szepietowska 2013, 2016; Gawda et al. 2017; GSass et al. 2013). Furthermore, a new approach to examining the words generated by subjects has been proposed here. This analysis is focused on reconstructing a semantic network of words reflecting the structure of emotion concepts. Words generated in affective verbal fluency tasks reflect the knowledge available to the subject relating to particular emotions. Generated words are not uttered accidentally; on the contrary they are related semantically to each other, words form semantic clusters, and the content of these clusters can be described (Berto and Galaverna 2016). The contents of semantic clusters are interesting if we want to understand the structure of emotional knowledge. A semantic network composed of semantic clusters reflects the structure of the concept. This approach and the way of assessing emotional concepts has been inspired by research on the organization of semantic knowledge based on the previous studies, such as a network approach based on verbal fluency data in assessment: the organization of the animal category (Goni et al. 2010), behavioral differences between healthy subjects, patients with cognitive impairment (Lerner et al. 2009), multidimensional approach to verbal fluency in patients with frontal and right lobe damage (Schwartz and Baldo 2001), organization of concepts based on material derived from the letter fluency (Schwartz et al. 2003), semantic memory in patients with Alzheimer’s disease (Chan et al. 1993), semantic memory organization in verbal fluency test “Human Body Parts” in patients with schizophrenia (Berto and Galaverna 2016). Although some studies carried out so far have used the qualitative analyses of Animals and letter categories of verbal fluency tasks, none of them was focused on emotional categories of verbal fluency.

The aim of the present studies is not to search for the prototype properties of the concept of love but to describe the structure of the concepts within love spectrum, represented by the categories of *liking, infatuation, love*, and its differentiation on the basis of linguistic material. To our knowledge, this type of analysis using language has not been presented in the literature. In order to describe the structure of the concepts from love spectrum, EVFT tasks were used as we were inspired by the previous findings (Gawda and Szepietowska 2013).

Here is an overview of the two studies. The analysis of the structure of emotional concepts can provide insight to emotional functioning of a human being. The knowledge about the structure and functioning of emotion concepts is of great importance for understanding the human psyche. That is why the project aiming to describe the structure of emotion concepts has been undertaken. The whole project focuses on five groups of emotional concepts relating to basic emotions such as joy, sadness, fear, anger, and love (Russell 2005). These are concepts located in the taxonomic hierarchy between the superior (e.g. emotion) and the subordinate level (e.g. panic, fear). The part of the project reported in this paper refers to concepts within the love spectrum. Two studies have been conducted. The purpose of the first one was to analyze love spectrum concepts with use of the new technique i.e. emotional verbal fluency test (EVFT). The second was intended to demonstrate and confirm the psychometric properties of the new method for describing love concepts. The commonly applied verbal fluency techniques are based on a semantic or phonemic principle. In contrast, far less attention has been paid to emotional verbal fluency tasks. Psychology is still facing a need to develop appropriate tools to assess emotional aspects. There is a scarcity of methods allowing us to study the structure of concepts. The first study (Study I) reported here was designed to identify semantic clusters from words generated during love spectrum tasks, and to examine their relationship with the triangular theory of love by Sternberg; it was also intended to test reliability and validity of EVFT. The reliability of EVFT was assessed with test–retest (after 2 months) correlations and with inter-judge correlations. Construct validity is demonstrated by correlations of EVFT with other verbal fluency tasks and WAIS-R Vocabulary subtest, while discriminant validity is shown by correlations with Sternberg Triangular Love Scale (1997) and The State Trait Anxiety Inventory. Study II was performed to verify the semantic clusters identified in Study I, and examine the relationship between the semantic clusters using another conception of romantic love i.e. love attitudes proposed by Lee (measured by the Love Attitudes Scale). In the two studies, verbal intelligence was controlled; Vocabulary subtest scores were used in the screening procedure to qualify participants without impairments in verbal comprehension, and then, in correlational analyses with regard to reliability testing. The main research questions focused on what kind of semantic clusters could be identified from words generated during EVF tasks related to *liking*, *infatuation*, and *love*. The next questions were: what are the differences in identified clusters between tasks related to *liking, infatuation*, and *love*, and what are the internal relationships (within the categories of *liking, infatuation, love*) between semantic clusters? Then, we wanted to find out whether these semantic clusters are related to love concept in the Triangular Theory of Love, and love attitudes by Lee. Finally, we wanted to see whether EVFT is reliable in terms of inter-rater agreement and test–retest correlations, whether EVFT is characterized by acceptable construct and discriminant validity, i.e. whether EVFT scores correlate with other fluency tasks, verbal intelligence, and STAI scores.

#### Hypotheses

Words generated by participants are semantically related and form semantic clusters. It is assumed that clusters reflect personal concept (lay concept) of liking, infatuation, and love. Emotional verbal fluency technique reveals lay-conceptualization of concepts within the spectrum of love. The components of the lay concept of love are consistent with the triangular theory of love; they correspond to intimacy, passion, and commitment. All lay concepts in the love spectrum contain these elements, however, to the different degree. We expect that the lay concepts of love also contain other elements such as aesthetic, existential, and temporal, because love is related to personal values and one’s personality as well as motivations. The assumption of the hypothesis is based on the previous findings showing that lay-people listed many more features of love than scientists (Fehr 1988; Fehr and Russell 1991; Gawda 2008). Finally, we expect that lay-concept of *liking, infatuation*, and *love* contains an aspect identified in the theory of love proposed by Lee, i.e. Storge, Eros, Ludus, Mania, Agape, and Pragma.

## Study I

### Method

#### Procedure

##### Instruction

The participants were asked to name, in 1 min, as many words as possible, to represent the emotional categories: liking, infatuation, love. All subjects were tested individually. All the words generated were recorded by the experimenter, counted for every participant and for every task. The participants were asked to name isolated words rather than phrases. The responses were recorded and then scored by the researchers and a trained researcher assistant. Every word named by the participants for the specific category: liking, infatuation, love, was treated as meeting the relevant criterion. The emotional verbal category is particularly subjective, and only perseverations/mispronounced words were treated as not meeting the criterion. Initially, a preliminary examination was carried out using the letter fluency technique and the verbal intelligence measurement. In order to estimate the IQ, the Vocabulary Subscale, part of the WAIS-R Intelligence Scale was used; it is a tool designed to assess verbal understanding, abstract thinking as well as the ability to classify concepts, and is an indicator of linguistic competence of the subject (Brzeziński et al. 2004).

In the above-mentioned study the experimental protocol was approved by the Local Ethics Committee of the Department of Pedagogy and Psychology of Maria Curie-Skłodowska University.

#### Participants

The study involved a sample of 340 subjects (180 women, 160 men) aged between 18 and 49 years (*M* = 25.00, *SD* = 3.9), right-handed, heterosexual adults, who were randomly selected to participate. They did not have any psychiatric, neurological, or somatic impairments, and were not addicted to drugs or alcohol (demographic and other data based on the screening questionnaire). All participants were native Polish speakers. They completed EVF tasks, other verbal fluency tasks, i.e. Animals, letter ‘k’ (the EVF tasks were always performed in the same order), as well as WAIS-R, and self-measures questionnaires.

#### Techniques

1. The Emotional Verbal Fluency Technique (EVFT): The instruction was as follows: “In a moment I will ask you to do a short task. You will have 1 min for this. Start. … And now, please, name within 1 min as many words as possible matching the category: liking. … Now, please name within 1 min as many words as possible for the category: infatuation. … finally, please name as many words as possible for the category: love”. For all the categories of tasks, i.e. liking, infatuation, love, the following semantic clusters (semantic cluster comprises at least two words spoken in succession and related semantically, e.g. words *men, women* = cluster ‘people’) have been identified: Passion aspects (*sex, excitement, date*); Intimacy aspects (*closeness, understanding, empathy*); Commitment aspects (*marriage, wedding*); Short-term temporal perspective (the use of words being temporal expressions indicating short duration, on the scale: 1 [no such words], 2 [such words appear, e.g. *fleetingness, moment*], 3 [there are many words indicating short duration]; Long-term temporal perspective (the use of words being temporal expressions indicating sustainability on the scale: 1 [no such words], 2 [such words occur, e.g., *forever*], 3 [there are many words indicating long duration]; Aesthetic aspect (number of words being aesthetic expressions, e.g. *beauty, charm*); Existential aspect (the use of words being existential terms on the scale: 1 [no such words], 2 [such words appear], 3 [there are a lot of words pointing to existential aspects: e.g. *life, meaning, value]*; Eros love features (e.g. *passion, sex, jealousy*); Ludus love features (e.g. *fun, pleasure, party*); Storge love features (e.g. *friendship, loyalty, gentleness*); Pragma love features (e.g. *decision, children, family*); Mania love features (e.g. *madness, insanity*); Agape love features (e.g. *dedication, devotion*). The analysis took into account correct responses after repetitions were excluded. The words generated in each of the tasks (liking, infatuation, love) were assigned to semantic clusters, and then the number of words in each cluster and/or assessment category was counted. Identification of clusters and their evaluation was performed by independent competent judges. The inter-rater agreement between judges’ assessments was very high (see Table 2).

A sample list of words generated by a participant: *happiness, joy, elation, delight, wedding, couple, reception, bride, groom, flowers, life, gifts, journey, happiness, contentment, rings, heart, forever.*

The coding system (also taking into account the semantic clusters):

- Number of words: 18
- Aspects of passion: 2 (*elation, delight*),
- Aspects of intimacy: 4 (*relationship, happiness, contentment, joy*),
- Aspects of commitment: 6 (*wedding, couple, reception, bride, groom, rings*),
- Short-term temporal perspective: 1 (no words related to this aspect),
- Long-term-temporal perspective: 2 (*forever*),
- Aesthetic expressions: 1 (*delight*),
- Existential aspects: 2 (*life, journey*),
- Eros aspects: 2 (*elation, delight*),
- Ludus aspects: 3 (*reception, gifts, flowers*),
- Storge aspects: 1 (*happiness, happiness, joy, contentment*),
- Pragma aspects: 1 (*couple*),
- Mania aspects: 0 (no words related to this),
- Agape aspects: 0 (no words related to this),

2. The Sternberg Triangular Love Scale (TLS) by Sternberg (1997). Based on this questionnaire three components of love were defined: Intimacy, Passion, and Commitment. The questionnaire consists of 36 items divided into three parts, each containing 12 questions and each measuring a different component. Consecutive items of the questionnaire are presented in a form of affirmative sentences describing one’s own beliefs and feelings towards a partner. These items each are rated on a seven-point scale, from ‘definitely no’ to ‘definitely yes’, a higher number marked by the respondent reflects his/her greater agreement with the given statement. The general score in each part is the sum of the points acquired for the items composing it. The psychometric properties of the Triangular Love Scale are very good (Sternberg 1997). Reliability of this scale in the present study was estimated by Cronbach’s alpha measures: Cronbach’s alpha for subscales: Passion = .87, Intimacy = .88, Commitment = .82.
3. The Wechsler Adult Intelligence Scale-Revised is a general test of intelligence, based on 11 subtests divided into two parts: verbal and performance. Vocabulary scores were first used in the screening procedure to classify and compare the candidates’ working memory and verbal comprehension, in order to select for the study only those subjects who presented no related impairments (Brzeziński et al. 2004). Then, these scores were used in the correlational analyses. Cronbach’s alpha in the present study for Vocabulary = .92.
4. The State Trait Anxiety Inventory. In this analysis the Trait/State Anxiety scores were considered. The Polish adaptation of the STAI consists of 20 statements describing emotional conditions. The respondents rated applicability of each statement to themselves on a 4-point scale: 1—rarely, 2—sometimes, 3—often, 4—usually. The reliability and validity of the STAI are very good (Wrześniewski et al. 2002). Cronbach’s alpha in the present study for trait anxiety = .88, for state anxiety = .87.

#### Statistical Analysis

First the descriptive statistics were computed (they are presented in the Table 1) in order to test distribution of variables and other properties of variables. Then, to assess reliability, Kendall’s coefficients of concordance (Kendall’s W) were computed between judges’ scores, and Pearson’s correlations were applied to perform test–retest reliability assessment. Construct validity was established with Pearson’s correlations between the EVFT scores and other verbal fluency scores, i.e. Animals and letter ‘k’. Similarly, Pearson correlations for the EVFT scores and the self-measures were used to assess discriminant validity. To show the structure of the concepts related to love spectrum, three hierarchical clustering analyses, using the squared-Euclidean distance and the nearest neighbour method, were conducted for the EVFT categories, i.e. *liking, infatuation, love*.

**Table 1:** Descriptive statistics

Variables	Liking (*n *= 340) *M (SD)*	Infatuation (*n *= 340) *M (SD)*	Love (*n *= 340) *M (SD)*
Correct responses	9.10 (4.81)	9.65 (4.44)	11.01 (5.01)
Number of errors	.01 (.03)	.03 (.04)	.06 (.05)
SC: Passion	1.94 (1.48)	4.77 (2.74)	3.04 (2.09)
SC: Intimacy	3.53 (2.80)	3.11 (2.66)	5.26 (3.220
SC: Commitment	2.14 (2.34)	2.38 (2.46)	4.39 (2.52)
SC: Short-temporal	1.10 (.37)	1.08 (.38)	1.02 (.21)
SC: Long-temporal	1.04 (.26)	1.11 (.44)	1.29 (.65)
SC: Esthetic	.21 (.46)	.36 (.58)	.22 (.49)
SC: Existential	1.01 (.19)	1.08 (.27)	1.32 (.61)
SC: Eros	1.79 (1.57)	4.30 (2.80)	2.40 (1.85)
SC: Pragma	1.04 (1.55)	1.00 (.00)	1.00 (.00)
SC: Ludus	1.55 (1.12)	1.19 (.95)	1.05 (.33)
SC: Storge	5.39 (3.10)	3.46 (2.26)	5.60 (3.09)
SC: Mania	1.16 (.70)	2.02 (2.04)	1.46 (1.53)
SC: Agape	1.23 (.97)	1.70 (1.59)	3.16 (2.57)

*SC* semantic cluster, *M* mean, *SD* standard deviation

### Results

#### Reliability

The reliability of the EVFT has been assessed with test–retest (after 2 months) Pearson’s correlations, and with inter-judge correlations (Kendall’s W tests were computed). Table 2 shows that the correlations between the judges’ assessments of different semantic clusters are very high (they range from .87 to .99). The correlations for test-retests (participants were examined again after a month) are also significant and high (they range between .76 and .92). This means that the criteria, such as number of words, errors, types of semantic clusters for indicator of EVFT are accurately defined and they are clear for independent judges.

**Table 2:** The inter-judge correlations (W-Kendall tests)

Variables	Liking (*n *= 340)	Infatuation (*n *= 340)	Love (*n *= 340)
Correct responses	.99***	.99***	.99***
Number of errors	.99***	.99***	.99***
SC: Passion	.98***	.98***	.98***
SC: Intimacy	.97***	.97***	.96***
SC: Commitment	.96***	.95***	.96***
SC: Short-temporal	.95***	.94***	.96***
SC: Long-temporal	.94***	.93***	.94***
SC: Esthetic	.87***	.91***	.87***
SC: Existential	.93***	.94***	.93***
SC: Eros	.98***	.98***	.98***
SC: Pragma	.93***	.92***	.93***
SC: Ludus	.92***	.91***	.90***
SC: Storge	.94***	.95***	.94***
SC: Mania	.91***	.90***	.89***
SC: Agape	.91***	.89***	.90***

*SC* semantic cluster

***p < .001

#### Validity

##### Construct Validity

Construct validity of EVF technique was demonstrated by Pearson’s correlations between the EVFT (semantic clusters and number of words in the categories of liking, infatuation, love) and other fluency tests (Animals, letter ‘k’). Table 3 shows there are many significant correlations (ranging from .37 to .44) which indicate correspondence between the number of words produced in EVFT task and in animals and letter ‘k’ tasks. This provides evidence for convergent validity of the EVFT. This means that the EVFT measures partly the same cognitive functions as the other verbal fluency tests and the WAIS-R Vocabulary subscale. This means that EVF is a measure of vocabulary and verbal intelligence.

**Table 3:** Correlations between the EVFT (number of correct responses) and other verbal fluency tasks (number of correct responses), and other measures (n = 340)

Variable	EVFT Liking (*n *= 340)	EVFT Infatuation (*n *= 340)	EVFT Love (*n *= 340)
VF: Letter k	.41***	.43***	.42***
VF: Animals	.42***	.44***	.44***
VOC	.37***	.36***	.34***
STAI-state	.11ns	.10ns	.11ns
STAI-trait	.18*	.09ns	.14*

*ns* non-significant, *EVFT* Emotional Verbal Fluency Technique, *VF* verbal fluency technique, *VOC* Vocabulary, subscale from WAIS-R, *STAI* results from the State-Trait Anxiety Inventory

*− *p* < .05; **− *p* < . 01; ***− *p* < .001

##### Discriminant Validity

To establish discriminant validity, two techniques were used: The Sternberg Triangular Love Scale (1997), and The State Trait Anxiety Inventory (Polish adaptation by Wrześniewski et al. 2002). In this analysis only the Trait/State Anxiety scores were considered. Pearson’s correlations were computed for the EVFT scores and the scores in the STAI and the TLS. The findings show that the number of words produced in the EVFT task does not correlate with the scores on the state and trait anxiety scales. Conversely, semantic clusters identified by the EVFT correlate with the TLS. More specifically EVFT Passion clusters correlate significantly with TLS Passion subscale; EVFT Intimacy clusters correlate with the scores in TLS Intimacy subscale, and EVFT Commitment clusters correspond to the scores in TLS Commitment subscale (see Table 4). This is evidence that EVFT semantic clusters enable measurement of similar aspects as those assessed by the TLS, i.e. a similar love component may be identified in the sequence of persons’ free recall related to the word ‘love’ and by the scores in the questionnaire constructed by Sternberg (1997). The existence of significant positive correlations between the EVFT and TLS, and a lack of correlations between the EVFT and the STAI confirm discriminant validity of the EVFT.

**Table 4:** Correlations between the EVFT (semantic clusters i.e. number of words in a cluster) and the TLS (*n* = 340)

EVFT	Semantic cluster	TLS Passion	TLS Intimacy	TLS Commitment
Liking	Passion	.14*	− .05ns	.07ns
	Intimacy	.04ns	.10ns	.10ns
	Commitment	.07ns	.18**	.27**
Infatuation	Passion	.83**	.10ns	.01ns
Love	Intimacy	.30**	.22**	.04ns
	Commitment	.28**	.26**	.05ns
	Passion	.31**	.11ns	− .04ns
	Intimacy	.13*	.71***	.42**
	Commitment	.11ns	− .01ns	.64***
Liking	Short-temporal	− .09ns	.02ns	− .03ns
Infatuation	Short-temporal	− .11ns	.00ns	.57**
Love	Short-temporal	.09ns	− .14*	− .01
Liking	Long-temporal	− .08ns	.09ns	.01ns
Infatuation	Long-temporal	− .01ns	.16*	.02ns
Love	Long-temporal	.15*	.15*	.39**

*p < .05; **p < . 01; ***p < .001

*ns* non-significant, *EVFT* Emotional Verbal Fluency Technique, *TLS* scores from the Triangular Love Scale

#### Standardization of the Technique: Normative Data

The normative data of the EVFT have been elaborated, as presented in Table 5. However, these normative data must be interpreted with caution due to the insufficiently large sample size. The normative data refer exclusively to Polish language.

**Table 5:** Normative data of the EVFT of ‘love’ category (*n *= 340)

Semantic clusters	Percentiles						
	5	10	25	50	75	90	95
Passion	1.00	1.00	1.25	2.00	4.00	6.00	8.00
Intimacy	1.00	1.00	2.00	5.00	9.00	10.00	10.00
Commitment	1.00	1.00	1.00	3.00	8.00	10.00	10.00
Short-term	1.00	1.00	1.00	1,00	1.00	1.00	1.00
Long-term	1.00	1.00	1.00	1.00	1.00	3.00	3.00
Esthetic	.00	.00	.00	.00	.00	1.00	1.00
Existential	1.00	1.00	1.00	1.00	1.00	2.00	3.00
Eros	1.00	1.00	1.00	2.00	3.00	5.00	6.95
Ludus	1.00	1.00	1.00	1.00	1.00	1.00	1.00
Storge	1.00	2.00	3.00	6.00	8.75	10.00	10.00
Mania	1.00	1.00	1.00	1.00	1.00	2.00	4.00
Pragma	.00	.00	.00	.00	.00	1.00	1.00
Agape	1.00	1.00	1.00	2.00	4.75	7.00	9.95

#### Hierarchical Clustering Analyses

At the start, the number of words generated in each category was compared. The participants generated approximately 10 words on average for each task category. For the liking category, people named fewer words while for the love category they named the largest number of words (see Table 1). There were significant differences between the number of words generated for liking and love (*t* = − 3.59, *p* < .001, *d* = .39), and the number of words produced in the category in infatuation and love (*t* = − 3.09, *p* < .01, *d* = .30). Interestingly, the number of words in each category did not correlate with the level of verbal intelligence determined using the Vocabulary subtest. Furthermore, the number of words generated by the participants did not correlate with age or sex. This means that the quantitative differences do not significantly impact the results obtained regarding the difference in the content of concepts which will be presented in subsequent step of analyses.

**Fig. 1:**
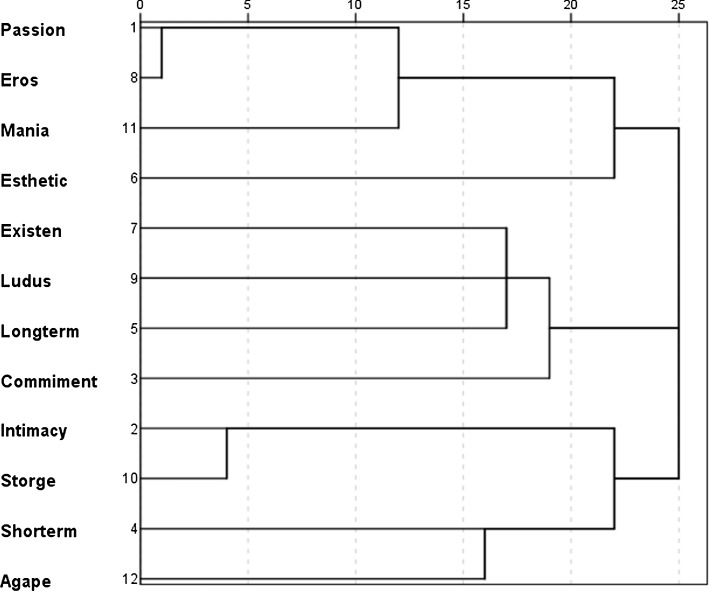
Hierarchical clustering: relationships between semantic clusters in the concept *love* (dendrogram)

Then, the semantic clusters in each category were compared. Cluster Pragma was excluded from the subsequent analyses because of the significant deviation from normal distribution. In the category *love* there were a lot of words related to commitment and intimacy and fewer related to passion. *Infatuation* contained more words/clusters related to aspects of passion and Eros type love (see Table 1). *Liking* encompassed more words/clusters related to intimacy and Storge type love. The concept of *liking* seems to be related to friendship and is different than the concepts of *love* or *infatuation*. Hierarchical clustering revealed that the love concept is formed of three main clusters similar to the component of love proposed by Sternberg (see Fig. 1). The first component/cluster contains passion aspects, Mania type love, Eros type love, and aesthetic aspects. This is a similar component to passion in the Sternberg’s theory of love. Interestingly, passion is perceived as a set of intense, even obsessive, feelings with associations to attractiveness. This means that attractiveness can be an essential aspect of passion. The second major cluster contains commitment, long-term temporal perspective, Ludus type love, and existential elements. It is similar to commitment in the Sternberg’s conception and is associated with long-term plans, some existential reflections, and overall positivity (which is connected with Ludus aspects). Finally, the third major cluster which can be named, i.e. intimacy, typically contains such element as intimacy, Storge type love, Agape type love. Interestingly, it also contains short-term temporal references which probably means that people do not believe that love may last for a long time.

**Fig. 2:**
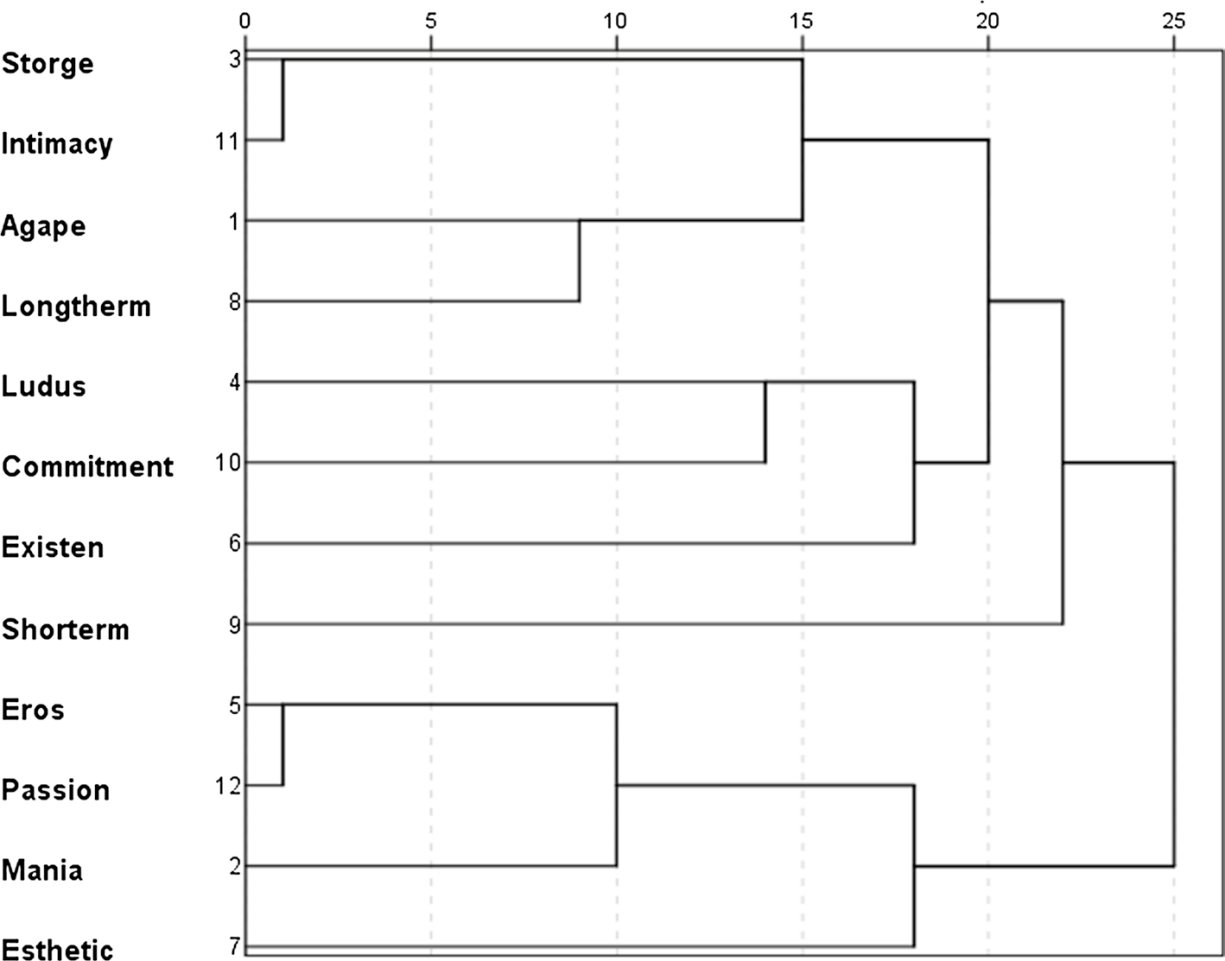
Hierarchical clustering: relationships between semantic clusters in the concept *infatuation* (dendrogram)

Hierarchical clustering for the category *infatuation* revealed that the structure of this concept is somewhat different than the love structure (see Fig. 2). The structure of the concept of infatuation is composed of two main clusters. The first one encompasses intimacy, Storge type love, Ludus type love, Agape type love, commitment, long-term temporal perspective, short-term temporal perspective, and existential aspects. It is a constellation of diverse elements showing that infatuation is perceived partly as friendship, a game-playing relationship, a serious relation, and partly it is seen in altruistic terms. It may have longer or shorter duration, and sometimes is associated with existential reflections. The second main cluster contains passion, Eros type love, Mania type love, and aesthetic aspects which reflects the essence of passion. The esthetic aspects are strictly related to the notion of passion. It manifests in lay-conceptualization of passion through references to attractiveness, i.e. people say *beauty, appeal, delight* when thinking about a beloved person and infatuation.

**Fig. 3:**
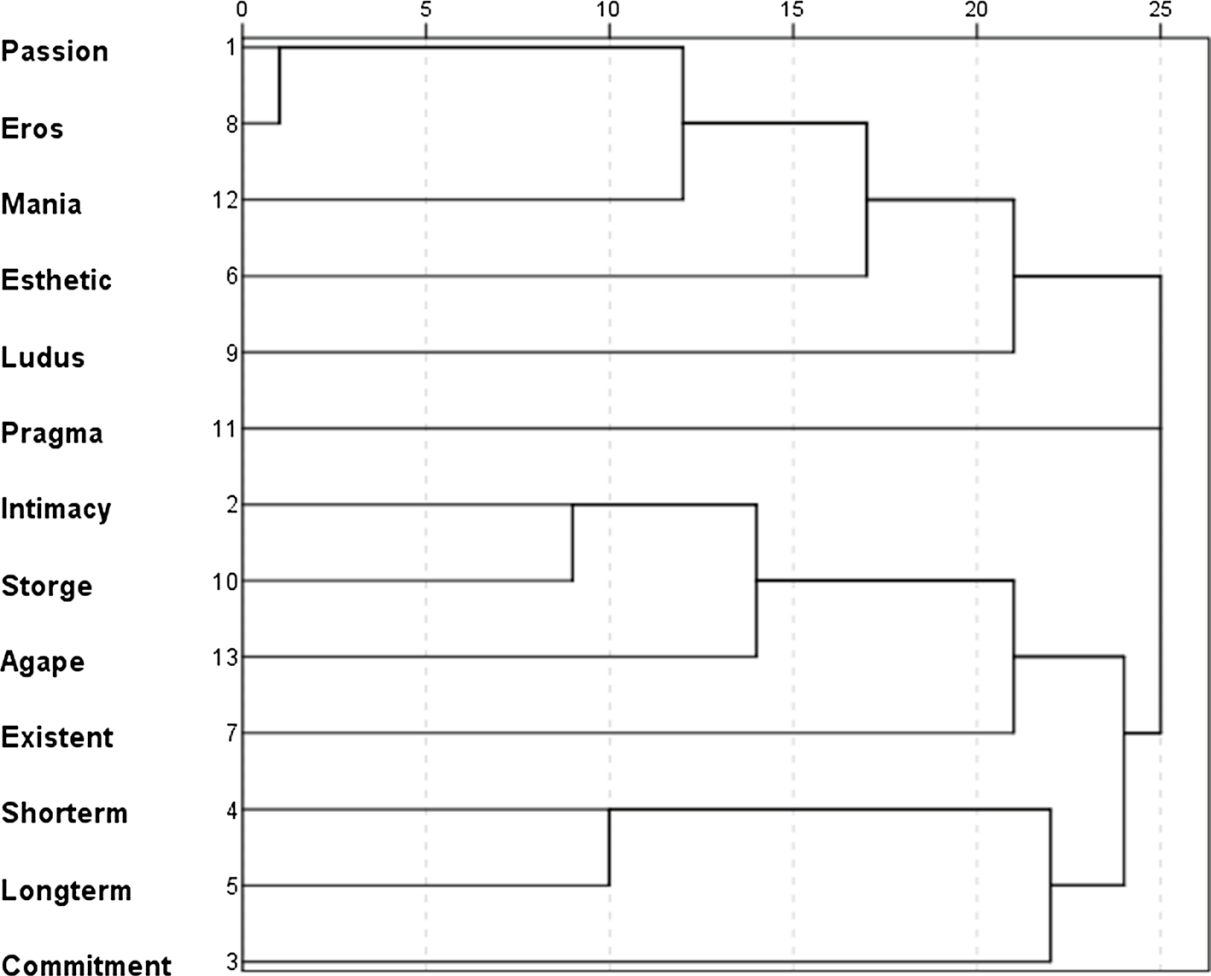
Hierarchical clustering: relationships between semantic clusters in the concept *liking* (dendrogram)

The structure of the semantic network of *liking* differs from the *love* and *infatuation* structures (Fig. 3). Hierarchical clustering reveals two main clusters (‘Pragma aspect’ cluster was included only in the case of the *liking* category): the first one encompasses components related to passion such as Eros type love, Mania type love, passion aspects, aesthetic aspects, and Ludus type love. The second main cluster contains many elements such as intimacy, Storge type love, Agape type love, existential aspects, short- and long-temporal perspectives, and commitment. This means that *liking* is perceived partly as friendship and can also be associated with sexual/passionate type of relationship. Interestingly, Pragma type love is unrelated to other aspects of the concept of liking. It is possibly due to the specificity of Pragma which is opposite to true friendship and not associated with passionate relationship.

## Study II

Aim: The second study was intended to examine validity of the EVFT in terms of identifying the six love attitudes proposed by Lee (and measured by the Love Attitudes Scale) from words generated by participants.

### Method

#### Participants

A sample of 190 participants (100 women, 90 men), between 18 and 50 years of age, were examined (M age = 25.00, SD = 3.57). All of them were right-handed, heterosexual adults; they did not have any psychiatric, neurological, or somatic impairments, and were not addicted to drugs or alcohol (demographic and other data based on the screening questionnaire). All participants were native Polish speakers. Participants completed the EVFT individually, other verbal fluency tasks, i.e. Animals, letter ‘k’, WAIS-R, and the Love Attitude Scale.

#### Procedure and Measures

The EVFT: the same procedure was applied as in Study I; the participants named all words coming to their mind representing the categories of liking, infatuation, and love. Then, to identify semantic clusters among the words produced by the subjects we used computer software constructed in order to accelerate the coding process and data analysis. The software, designed to count words, is based on Excel formula. First, all words semantically associated with passion, intimacy, and other clusters were entered into the database of the software, then a special mathematical formula was applied to enable the system to count all the words representing the specified clusters. Subsequently, a list of words produced by a subject was uploaded into the software. As a result the software produced numerical scores for all the types of clusters which were programmed. In terms of its functionalities, the software is similar to the LIWC (Linguistic Inquiry and Word Count) by Pennebaker, Booth, Boyd, and Francis (2015); it counts the number of words in the following clusters: Passion aspects, Intimacy aspects, Commitment aspects, Eros type, Mania type, Storge type, Agape type, Pragma type, and Ludus type. The coefficients of equivalence between the software scores and independent judges’ scores are very high (k ranges between .98 and 1.00).

Next, participants completed the Love Attitude Scale (LAS), a 42-item questionnaire designed to measure attitudes towards love. The questionnaire combines attitudes towards one’s current/recent/hypothetical partner with attitudes related to love in general. The scale is broken into six subscales (seven items each), representing different love styles: Eros (passionate love), Ludus (game-playing love), Storge (friendship love), Pragma (practical love), Mania (possessive, dependent love), Agape (altruistic love). Participants respond to each item using a 5-point scale: 1 (strongly agree), 2 (moderately agree), 3 (neutral), 4 (moderately disagree), 5 (strongly disagree). In three prior studies, the average alpha coefficient was found to be .80 (Hendrick and Hendrick 1989; Hendrick et al. 1998). The consistency of this scale is reported as follows: for Eros (.71), Ludus (.75), Storge (.84), Pragma (.82), Mania (.71), and Agape (.84). In the present study Cronbach’ alpha values are also appropriate: for Eros = .76, Ludus = .78, Storge = .85, Pragma = .68, Mania = .79, Agape = .88.

### Results

First, the numbers of words generated by the participants in the categories of *liking*, *infatuation*, and *love* were examined in relation to the scores in the other fluency test (Animals, letter ‘k’); for this purpose Pearson’s correlations were computed. Many significant correlations (ranging between .42 and .45) confirmed construct validity of the EVFT. This provides evidence for convergent validity of the EVFT, like in the first study. Then, correlations between EVFT and WAIS-R Vocabulary scores were computed. Significant correlations (ranging from .37 to .38) confirmed that the number of correct responses in EVFT is a measure of similar cognitive functions as those assessed by the Vocabulary subscale of the WAIS-R. Then, in order to establish discriminant validity, the LAS was used. Pearson’s correlations were computed for EVFT scores (semantic clusters Eros, Storge, Ludus, Mania, Pragma, Agape) and the scores in the LAS. The results show that the number of words in the semantic clusters identified by the EVFT correlate with the LAS (see Table 6). In the category of words related to liking, the clusters named Eros correlate significantly with LAS Eros and Mania subscales, Storge clusters correlate with LAS Storge subscale. No significant correlations were found between the LAS scores and clusters named Ludus, Pragma, and Agape. This may be due to the infrequent listing of words related to these aspects in the *liking* category. As regards the *infatuation* category, the scores in LAS Eros subscale correlate significantly with EVFT Eros and Mania clusters, those in LAS Storge subscale correlate with EVFT Storge clusters, the LAS Mania subscale correlates with EVFT Eros and Mania clusters; the correlations identified in the case of Ludus, Agape, and Pragma clusters were not significant which is potentially to due to the low number of words in these clusters in the *infatuation* category.

**Table 6:** Correlations between the EVFT (semantic clusters i.e. number of words in a cluster) and the LAS (*n* = 190)

EVFT	Semantic cluster	LAS Eros	LAS Storge	LAS Ludus	LAS Mania	LAS Pragma	LAS Agape
Liking	Eros	.32***	.11ns	.02ns	.18*	.03ns	.01ns
	Storge	.03ns	.17*	.01ns	.00ns	.02ns	.06ns
	Ludus	.11ns	.12ns	.13ns	.10ns	.04ns	.05ns
	Mania	.09ns	.02ns	.06ns	− .07ns	.07ns	.09ns
	Pragma	.02ns	.01ns	.02ns	.02ns	.01ns	− .02ns
	Agape	.07ns	.01ns	.07ns	.07ns	.02ns	.03ns
Infatuation	Eros	.82***	.13ns	.09ns	.24**	− .03ns	− .00ns
	Storge	.12ns	.21*	.01ns	.07ns	.00ns	.01ns
	Ludus	.11ns	.01ns	.03ns	.09ns	.01ns	.05ns
	Mania	.34**	.02ns	.06ns	.26**	.03ns	.04ns
	Pragma	.01ns	.00ns	.00ns	.03ns	.00ns	.02ns
	Agape	.00ns	.10ns	− .02ns	.02ns	.02ns	.02ns
Love	Eros	.56***	.11ns	.10ns	.17*	.07ns	.02ns
	Storge	.04ns	.22*	.12ns	.02ns	.03ns	.05ns
	Ludus	.13ns	12ns	.23*	.02ns	.01ns	.00ns
	Mania	.12ns	.01ns	.02ns	.25**	.04ns	− .06ns
	Pragma	.01ns	.01ns	.04ns	.00ns	.15*	.16*
	Agape	.02ns	.02ns	.03ns	.01ns	.10ns	.20*

*p < .05; **p < .01; ***p < .001

*ns* non-significant; *EVFT* Emotional Verbal Fluency Technique, *LAS* scores form the Love Attitude Scale

As for the *love* category, many significant correlations were found: LAS Eros subscale correlates with EVFT Eros cluster, LAS Storge subscale with EVFT Storge cluster, LAS Ludus subscale with Ludus cluster, Mania subscale with EVFT Eros and Mania clusters, Pragma subscale with Pragma semantic cluster, and finally LAS Agape subscale with EVFT Agape cluster. Many of these correlations are low, one is moderate. In general they confirm that semantic clusters identified taking into account the words generated by the subjects during the EVFT *love* task carry similar meanings as the subscales of the LAS. This shows that EVFT enables measurement of similar aspects as the LAS, i.e. similar love attitudes may be identified by the free-recall task *love* and the LAS questionnaire. These positive significant correlations between the EVFT and the LAS confirm validity of the EVFT.

## General Discussion

The presented findings on the EVFT, i.e. in the *liking*, *infatuation*, and *love* tasks, have shown that this is a reliable measure across raters and time. The types of semantic clusters identified based on these EVFT are precisely defined and they are clear for independent judges. Likewise, construct validity of these EVF tasks was demonstrated, and this suggests that EVF tasks refer to similar cognitive processes, corresponding to those measured by other verbal fluency tests. Furthermore, the documented links between other measures, such as the TLS and the LAS, and a simultaneous lack of correlations between the STAI and the EVF scores, confirmed discriminant validity the EVF tasks. It can be concluded that emotional verbal fluency tasks partly measure the same verbal-cognitive functions as the other verbal fluency tests and the WAIS-R Vocabulary subscale. This means that EVF tasks are a measure of vocabulary and verbal intelligence. This conclusion is consistent with other findings regarding emotional verbal fluency technique (e.g. Abeare et al. 2017).

Furthermore, the novel and creative aspect of qualitative data analyses with respect to EVF performance has been developed based on the previous propositions of multidimensional approach/correspondence analysis/network approach to verbal fluency, e.g. Berto and Galaverna (2016), Goni et al. (2010), Lerner et al. (2009), Schwartz and Baldo (2001) and Schwartz et al. (2003). This type of linguistic material analysis brings forward a new perspective on cognitive psychology of emotions. The presented qualitative examination of semantic clusters identified in the categories of *liking, infatuation,* and *love* allows describing the structure of these emotion concepts. The hierarchical cluster analyses revealed the structure of the concepts in the love spectrum. The three concepts of *liking*, *infatuation*, and *love* are different in terms of their structure, however, they are similar as they belong to the same spectrum. Reconstructed from the linguistic material, the semantic network named *liking* is associated with friendship, Storge type love, positive attitude, calmness, attachment, and gentleness. This may contain elements of passion which potentially means that *liking* can transform into *infatuation*. This lay-concept of *liking* is a mental representation of an easygoing and positive friendly relationship. It is consistent with Niedenthal’s statement that mental representations contain categories, objects, situations, and activities. The lay-concept of *liking* encompasses typical information on a close friendly relation, people involved in this relation, typical activities such as communication, meetings, talking, confessions. It also contains typical emotions and behaviours, such as joy, satisfaction, contentment (Niedenthal 2008; Shaver et al. 1987).

Another lay-concept described here is *infatuation*. In its structure the essential components is Eros type relationship. Lay people perceive *infatuation* as a constellation of intense emotion, sexual attraction, romance, physical attraction, sexual consummation, jealousy, and delight (Sternberg 1997, Hatfield and Rapson 1987, 1996). The concept of *infatuation* is almost identical with the concept of passionate love (Hatfield and Rapson 1987, 1996).

In lay concept of love, intimacy and Storge love type play essential role. Love is perceived as harmonious and intimate relationship. This is consistent with scientific theories e.g. proposed by Fehr (1988, 2006) and Shaver et al. (1987) who argue that love plays fundamental role among emotion concepts and its structure is prototypical. The structure of *love* concept contains emotions reflecting, for instance, atmosphere of openness, a sense of security, the relationship between people expressing they are liked, loved, need each other, and a feeling that people in this relationship agree with each other. As presented here, the structure of *love* concept transpiring from emotional verbal fluency is similar to the findings of Shaver et al. (1987). They reported a hierarchical cluster analysis of 135 emotion terms and established that there are three types of love: affection, lust, and longing. ‘Affection’ consists of adoration, love, fondness, liking, attraction, caring, tenderness, compassion, and sentimentality. This is similar to a notion of companionate love and our findings correspond with this. The cluster which by Shaver and associates was labelled ‘lust’, consists of arousal, desire, lust, passion, and infatuation, therefore it resembles our cluster of ‘passionate love’ containing Eros type love, passion, Mania type and aesthetic aspects. Finally, the cluster referred to as ‘longing’ consists only of the term longing according to Shaver et al. (1987). Although we did not identify such a semantic cluster, the corresponding notions may be incorporated in other elements of love structure, such as existential aspects. The latter semantic cluster contains existential references such as *lifetime, existence, time, to pass away*. With regard to the manifestations of love, cognitive and emotional aspects are shown (Fehr 1988; Fehr and Russell 1991; Shaver et al. 1987), such as difficulties with concentration, being captivated by the loved one, a strong feeling of happiness and joy, and a series of behaviours expressing a need to be together. The structure of the concept of *love* identified using the emotional verbal fluency technique is also charged with emotional elements, which is consistent with other scientific findings (Sternberg and Weis 2006). The structure of *love* concept is not only related to companionate love type, it contains elements of passion as well. The essential aspect of *love* concept also comprises the notion of commitment, not only according to Sternberg’s conception (1986, 2006) but also in lay-people conceptualization of romantic love, as shown by the emotional verbal fluency technique. The notion of commitment contains information related to decisions and long-term plans associated with a beloved person, which was illustrated by the present findings. On the contrary, intimacy is connected with short-term temporal perspective which potentially means that partners share the information and emotions for a limited duration of time. Perhaps the element of Ludus love in this configuration refers to the belief that a relationship must be pleasant, and greatly satisfying for the individuals involved. References to the unconditional aspect of feelings incorporated in the concept of Agape love, and dedication associated with unconditional adoration of a person, are also perceived by lay people as important elements of love concept, and intimate relationships in particular.

In the structure of *love* concept, an interesting configuration is formed by passion, Eros type and Mania type love, as well as aesthetic aspects. This is most likely associated with the specific concept of beauty. Admiration is embedded in love, but it is not only about the physical attractiveness of the partner, which would be typical of passion (Lieberman and Hatfield 2006). Personal concept of beauty is associated with the emotional bond with the partner and is formed in the context of emotional closeness; it therefore reflects an admiration for the partner’s skills, his/her character, kindness, or it is an expression, in aesthetic terms, of the emotional relationship. In general, we may agree with a statement by Fehr (1988) saying that lay people possess larger knowledge of emotional concepts within love spectrum than scientists propose. She argued that lay people know more subtypes of love or types of love than stipulated by the theories. Scientists have typically distinguished two, three, or six types of love (Fehr and Russell 1991). Lay people possess large knowledge about love; their concepts of love contain different type of information, in such categories as erotic, passionate, pragmatic, altruistic, ludic, manic, romantic, brotherly love, charismatic, discipleship love, infidelity, infatuation, and parent-infant love. Lay people perceive love and concepts within love spectrum in general terms, as defined by Johnson-Laird and Oatley (1989), i.e. as an expression of the capacity “to experience internal happiness in relation to an object, or person, who may also be the object of sexual desire” (p. 60). All these aspects can be analyzed using emotional verbal fluency techniques.

The above analyses of the cognitive structure of emotion concepts address connectionist models of semantic knowledge. Words generated freely during emotional verbal fluency tasks are interconnected semantically, conveying information on the structure of semantic networks of ‘liking’, ‘infatuation’, and ‘love’. This spontaneous grouping of words can be explained in terms of a “spreading activation” model (e.g. Anderson and Pirolli 1984). This model assumes that words as represented in mind as interconnected nodes; concepts sharing many attributes are strongly connected, and activation of a given word automatically activates local associations, a local network (Schwartz et al. 2003). This means that activity within semantic networks spreads instantaneously between related nodes. As a result sets of interconnected words are generated during verbal fluency tasks. This semantic effect was observed in the present study where a lot of related words were produced by the subjects performing EVFT related to liking, infatuation, and love. The “spreading activation” model explains how the automatic semantic effect influences emotional verbal fluency performance (in another study, semantic effect was found for letter verbal fluency, Schwartz et al. 2003). This is due to two factors, first, semantic proximity of words in a given network (it is easier to retrieve closely related words than those which are distant from each other), and second, time pressure which limits the search process to related words. From the methodological perspective, the way of assessing proximity between words in hierarchical clustering or multidimensional scaling reflects this semantic connectivity. Thus, the presented method of analysing the relationships between words produced in course of an EVF tasks, makes it possible to describe their semantic relationships and, consequently, the cognitive structure of emotion concepts. EVFT allows to study other emotions. The present paper presents a part of a large project focused on the description of many emotion concepts. All the emotion concepts representing the basic level of categorization according to Shaver et al. (1992) have been studied in this project, i.e. love, fear, sadness, anger, and joy spectrum. The results of EVFT related to other emotions, such as fear, sadness, joy, and anger, are also interesting and promising. EVFT makes it possible to describe the structure of these concepts and show their differentiation (Gawda 2017). For instance, it has been found that the structure of the lay-concepts in the *fear* spectrum is varied and this diversity is associated with age, sex, trait anxiety, or mental rigidity. This means that men and women, low- and high dogmatic, as well as low- and high-anxious individuals differ in terms of the structure of the fear concept. Age modifies the structure of the concepts within fear spectrum (Gawda 2017; Gawda and Szepietowska 2016). In summary, this suggests that the structure of the concepts of love, fear, sadness, anger and other emotions partly reflects individuals’ subjective experiences. The structural components of the emotional concepts identified by examining the material collected using EVFT are in line with the existing scientific approaches indicating that emotional concepts include, for instance, interoceptive data, exteroceptive data such as visual, auditory, and olfactory information, specific due to the modality, and regarding objects and contexts in which these objects are used (Barrett 2014; Wilson-Mendenhall et al. 2013), as well as data on activities typical for given emotion categories (Oosterwijk et al. 2014).

To conclude, in view of the fact that there is a scarcity of methods allowing to study the structure of concepts, the new method referred to as emotional verbal fluency technique is of value. Narrative techniques can be used to examine emotions and emotion concepts, however, narrative analyses are considered to be labor-intensive, long lasting, and not fully objective. The EVFT presents a number of advantages. A participant needs just a few minutes to complete the EVFT (one task takes 1 min). The procedure required for performing an analysis of the generated words is less time-consuming compared to narrative analyses and because of the computer aid software. In summary, EVFT is less troublesome than narrative tasks for the examined person and for the psychologist. It also seems to be a more objective measure because we may use computer software.

### Limitations

The proposed procedure of analyses of qualitative data obtained during emotional verbal fluency tasks is a time-consuming process, however, it is less labor-intensive, shorter, and simpler to execute than, for instance, analyses of material collected from narrative techniques. In order to accelerate examination of the linguistic material it is necessary to develop better computer software.

### Future Directions

The aim of future studies is to describe other emotion concepts, by using EVFT, and develop related software to support the coding procedure as well as to facilitate replication studies.
